# Nonlinear model of infection wavy oscillation of COVID-19 in Japan based on diffusion kinetics

**DOI:** 10.1038/s41598-022-23633-8

**Published:** 2022-11-10

**Authors:** Tatsuaki Tsuruyama

**Affiliations:** 1 Department of Physics, Graduate School of Science, Tohoku University, Sendai, 980-8578 Japan; 2 Department of Drug Discovery Medicine, Medical Innovation Center, Kyoto University Graduate School of Medicine, Kyoto, 606-8501 Japan; 3 Tazuke Kofukai Medical Research Institute, Kitano Hospital, Osaka, 530-8480 Japan; 4 Department of Molecular Biosciences, Radiation Effects Research Foundation, Hiroshima, 732-0815 Japan

**Keywords:** Computational biology and bioinformatics, Preventive medicine, Air microbiology, Systems biology, Computer modelling, Computer science, Nonlinear dynamics, Numerical simulations, Oscillators, Population dynamics

## Abstract

The infectious propagation of SARS-CoV-2 is continuing worldwide, and specifically, Japan is facing severe circumstances. Medical resource maintenance and action limitations remain the central measures. An analysis of long-term follow-up reports in Japan shows that the infection number follows a unique wavy oscillation, increasing and decreasing over time. However, only a few studies explain the infection wavy oscillation. This study introduces a novel nonlinear mathematical model of the new infection wavy oscillation by applying the macromolecule diffusion theory. In this model, the diffusion coefficient that depends on population density gives nonlinearity in infection propagation. As a result, our model accurately simulated infection wavy oscillations, and the infection wavy oscillation frequency and amplitude were closely linked with the recovery rate of infected individuals. In conclusion, our model provides a novel nonlinear contact infection analysis framework.

## Introduction

Accurate predictions of new SARS-CoV-2 infections are essential to public health responses, such as restrictions on behaviour, isolation of the infectious individual, and effective allocation of medical resources. In Japan, the  number of new cases of infection has been reported, with the observation of infection wavy oscillation curves at least six times in the past 2 years^1^. Several models of infection transmission have been identified, including the SEIR model^2,3^. These models consist of three or four types of individuals: (1) *Susceptible* (*S*), an individual without immunity; (2) *Exposed* (*E*), the infectious individual during the incubation period; (3) *Infectious* (*I*); and (4) *Recovered* (*R*)(immunity acquirer)^4^. The simplest mathematical model of infectious disease transmission is the SIR model, which does not consider the Exposed group^5–7^. The SEIR model is easily understood by considering the population transition process using differential equations^1,8,9^. Its plot can predict chaotic oscillations of the population number^10,11^. However, the observed oscillation in these models is highly complicated, and it is difficult to produce the long-term oscillation of the number of infected people, which regularly increases and decreases in Japan.

In this study, we designed a nonlinear model explaining the infection wavy oscillation curve in reference to the SEIR model. We aimed to predict the infection number data to plan prevention measures against disease propagation. We applied the macromolecular diffusion theory to model infection transmission. Macromolecules modify or are modified by each other during diffusion by interaction with other macromolecules in the reaction system, and the diffusion process is a rate-limiting step. Analysis of the macromolecule diffusion requires physicochemical and hydrodynamic theory^2,3^. In the previously reported SIR/SEIR model, the kinetic equation includes *f*(*X*_*i*_; *S, I, R*, (*E*)) that consists of products of population densities *X*_*i*_ = *S, E, I,* or *R*, and diffusion item of the individuals, commonly written based on the macromolecule dynamics as^10,12,13^:

1 $$\frac{d{X}_{i}}{dt}=f({X}_{i}, {X}_{i}{X}_{j};{X}_{i},{X}_{j}=S, E, I, R)+{D}_{{X}_{i}}{\nabla }^{2}{X}_{i}$$

Equation (1) is a standard SEIR model, if diffusion terms are not considered. ${D}_{{X}_{i}}$ represents the diffusion coefficients of *X*_*i*_. We hypothesised that the diffusion process was a rate-limiting step. In this case, as described later, the diffusion coefficient, ${D}_{{X}_{i}{X}_{j}}$, depends on the population density^2,4,14–16^.

2 $$\frac{d{X}_{i}}{dt}={k}_{{X}_{i}{X}_{j}}{D}_{{X}_{i}{X}_{j}}({X}_{j}=S, E, I,R){X}_{i}{X}_{j}+{k}_{{X}_{i}}{X}_{i}+{k}_{{X}_{j}}{X}_{j},$$

where ${k}_{{X}_{i}{X}_{j}}$, ${k}_{{X}_{i}}$, and ${k}_{{X}_{j}}$ represent kinetic coefficients. We noted this diffusion dependency on the population density and performed numerical calculations to simulate infectious wavy oscillations. Subsequently, we performed numerical calculations using the kinetic model equations (Eq. 2) to simulate infectious wavy oscillations.

## Results

### Exposed, cluster, and infectious model

A three-individual model was considered with an exposed individual (*X* = *E*), an individual in the cluster (*W*), and infectious individual (*Z* = *I*) coexisting. Because the sum of the individuals, *E* + *W* + *I,* remained constant, the kinetics are attributed to a two-parametric analysis. In the present study, we summarised the infection transmission process as follows:

1. Individuals achieve an active infectious state by exposure to coronaviruses or by interaction with the exposed or infectious individual by contagion.
2. Individuals recover through therapeutic interventions supplied continuously and externally.
3. Individuals diffuse by the density gradient following an interaction with an exposed or infectious individual.

**Figure 1:**
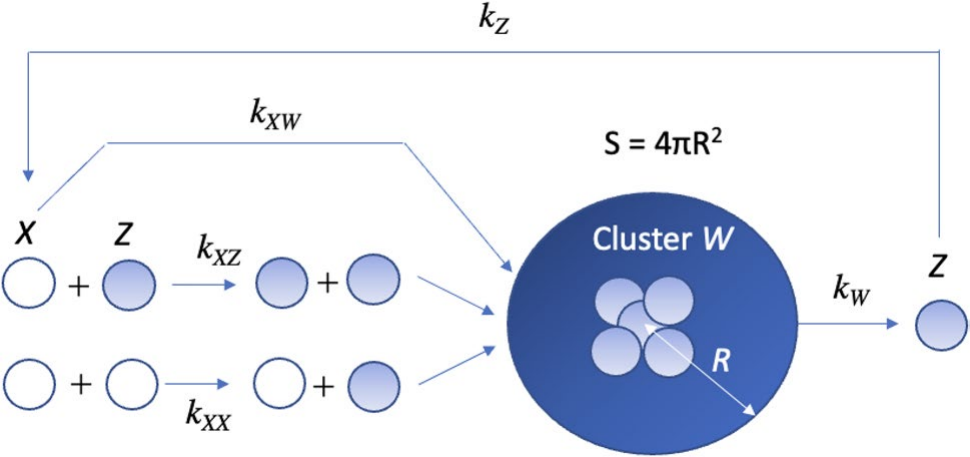
Scheme of infection transmission. Individual globules represent an “Exposed” *X*, an “Infectious” Z, and “Cluster” *W*. The recovery factor *P* supply is kept constant, and *Z* is released continuously from *W*. The differential equations are given in Eqs. (20)–(22). *R* indicates the cluster size. *k*_*XW*_, *k*_*Z*_, *k*_*WZ*_, *k*_*XX*_, and *k*_*XZ*_ represents the kinetic coefficients in Eqs. (20)–(22).

Individual diffusion was a rate-limit process because of the slow rate^2,4,16^ and calculated *v*_*XW*,_ using the interaction rate of an *X* to the cluster of infectious individuals, *W*, and the flux per unit area of the cluster, *J*. The interaction area can be determined by the "cluster size" *R* (Fig. 1). The interaction rate is given by:

3 $$v_{XW} = 4\pi R^{2} J$$

When Fick's first law holds, the flux *J* is given *R*, and *D*_*X*_, the diffusion coefficient of *X*:

4 $$J={D}_{X}{\left(\frac{dX}{dr}\right)}_{r=R}={D}_{X}\frac{X}{R}$$

By substitution of (3) into (4), the rate of contagion *v*_*X*_ is given as

5 $${v}_{X}={\int }_{0}^{R}4\pi {R}^{2}{J}dR=4\pi R{D}_{X}X$$

Accordingly, the global flow of all *X* to *W* is 4*πRD*_*X*_* XW*. Similarly, the flow of all *W* to *X* is 4*πRD*_*W*_*WX* where *D*_*W*_ denotes the diffusion coefficient of *W.* Further, using the sum of the diffusion coefficients of *X* and *W,* the average diffusion coefficient can be written as *D*_*XW*_ = (*D*_*X*_ + *D*_*W*_)/2*.* The addition rate *v*_*XW*_ of *X* to *W* is given using an arbitrary coefficient *k*_*XW*_ :

6 $${v}_{XW}= 4\pi R{D}_{XW}XW={k}_{XW} {D}_{XW}XW$$

where

7 $${k}_{XW} = 4\pi R$$

Similarly, *Z* leaves the cluster to freely diffuse and becomes active for the infection; the kinetic rate is given using *D*_*WZ*_*,* the diffusion coefficient:

8 $${v}_{WZ}= {k}_{WZ} {D}_{WZ}W$$

*D*_*WZ*_ = (*D*_*W*_ + *D*_*Z*_)/2*.* In addition, the interaction (contagion) rates between *X* and *X*, and *X* and *Z* are denoted by *v*_*XX *_and *v*_*XZ*_, respectively. Using their kinetic coefficients, *k*_*XX*_ and *k*_*XZ*_, the kinetic rates are given by *v*_*XX*_ = *k*_*XX*_* D*_*XX*_* X*^*2*^ and *v*_*XZ*_ = *k*_*XZ*_* D*_*XZ*_* XZ* = *k*_*XZ*_ (*D*_*X*_ + *D*_*Z*_*)/2 XZ*, respectively*.*

When the infectious population density is sufficiently small, individuals may diffuse freely within a sufficiently large space, and the diffusion coefficient can be regarded as a constant. Here, the fluctuation of the population density is set as

9 $$X={X}^{st}+x,$$

10 $$W={W}^{st}+w,$$

11 $$Z={Z}^{st}+z.$$

*X*^*st*^ and *Z*^*st*^ denote the exposed and infectious population density at the steady state, respectively. *x* = *x*(*t*)*, w* = *w*(*t*), and *z* = *z*(*t*) denote the population density change from the steady state.

The diffusion coefficient ${D}_{{X}_{i}}$ of the *i-*th individual, *X*_*i*_, is given using coefficients *a*_*i*_ representing a dependency on the density fluctuation of the individual, *x*_*i*_ (*x*_*1*_ = *x, x*_*2*_ = *w, x*_*3*_ = *z*)^2,17,18^:

12 $${D}_{{X}_{i}}={{D}_{{X}_{i}}}^{st} (1-\sum_{i=1}^{3}{a}_{i}{x}_{i})$$

${{D}_{{X}_{i}}}^{st}$ is the diffusion coefficient when the contribution of *x*_*i*_ is negligible at the steady state. A negative sign indicates that the diffusion coefficient decreases with increasing polymer concentration. The signs of *a*_*i*_ can be either positive or negative. Below, we applied the diffusion theory for modelling the infection transmission by replacing the macromolecule concentration with the population density^2,14,15,17,19^. According to Eq. (12), the diffusion coefficients of *X* and *Z* are given by applying the above formula to a mix of two individuals, *X* and *Z,* when these are sufficiently low:

13 $${D}_{X}={{D}_{X}}^{st}(1-{a}_{X}x-{a}_{Z}z)$$

14 $${D}_{Z}={{D}_{Z}}^{st}\left(1-{b}_{X}x-{b}_{Z}z\right)$$

Because the cluster *W* diffusion rate is small, *w* and *D*_*W*_ were neglected above. *D*_*X*_^*st*^ and *D*_*Z*_^*st*^ denote the diffusion coefficients of individuals *X* and *Z* at steady state, respectively, and are constants.

### Kinetic equation of infection model

First, *X* can irreversibly be in contact with the cluster, a part of which can be a member of the cluster:

15 $$X+W\leftrightarrow W:{k}_{XW}$$

Subsequently, *Z* leaves the cluster to be free:

16 $$W\to Z:{k}_{WZ}$$

Furthermore, *Z* is treated to recover to *X*:

17 $$Z + P \to X:k_{Z}$$

*P* represents the recovery factor. In addition, the *X* interaction is promoted to be infectious:

18 $$X + X \to X + Z:k_{XX}$$

Furthermore, *X* contagion with *Z* is promoted to be Z:

19 $$X + Z \to 2Z:k_{XZ}$$

The contagion scheme is shown in Fig. 1. Summarising Eqs. (15)–(19), the kinetic equations of *X*, *W*, and *Z* are written as follows:

20 $$\frac{dX}{dt}=-{k}_{XW}{D}_{XW}WX+{k}_{Z}PZ-{k}_{XX}{D}_{XX}{X}^{2}-{k}_{XZ}{D}_{XZ}XZ$$

21 $$\frac{dW}{dt}={k}_{XW}{D}_{XW}WX-{k}_{WZ}{D}_{W}W$$

22 $$\frac{dZ}{dt}={k}_{W}{D}_{WZ}W-{k}_{Z}PZ+{k}_{XX}{D}_{XX}{X}^{2}+{k}_{XZ}{D}_{XZ}XZ$$

Further, for simplicity, Eqs. (20) and (22) are given by replacing the kinetic coefficients with arbitrary coefficients:

23 $$\frac{dX}{dt}=-{h}_{1}WX+pZ-{h}_{4}{X}^{2}-{h}_{5}XZ$$

24 $$\frac{dZ}{dt}={h}_{2}W-pZ+{h}_{4}{X}^{2}+{h}_{5}XZ$$

Here, *k*_*XW*_*D*_*XW*_ = *h*_*1*_, *k*_*WZ*_W = h_2_, *k*_*XX*_*D*_*XX*_ = *h*_*4*_*, k*_*XZ*_*D*_*XZ*_ = *h*_*5*_, and *p* = *k*_*Z*_* P.* In the following, *p* will be called a recovery rate factor in the following. Accordingly, the kinetic coefficients *h*_*2*_, *h*_*4*_ and *h*_*5*_ are proportional to the diffusion coefficients that depend on the population density. Setting the right-hand sides of Eq. (23) and (24) equal to zero, we have the concentration of *X* and *Z* at a steady state, *X*^*st*^ and *Z*^*st*^.

25 $${X}^{st}=\frac{{h}_{2}}{{h}_{1}},{Z}^{st}=\frac{{h}_{2}({h}_{2}{h}_{4}+{{h}_{1}}^{2}W)}{{h}_{1}({h}_{1}p-{h}_{2}{k}_{5})}$$

Substitution of Eqs. (9), (10), and (11) into Eqs. (23) and (24), respectively, and altering *h*_*1*_, *h*_*4*_, and *h*_*5*_ to *h*_*1*_ − *ax* + *bz*, *h*_*4*_ − *cx* + *dz*, and *h*_*5*_ − *ex* + *fz*, respectively, we have

26 $$ \begin{aligned}\frac{dx}{dt}&=-\left\{W\left({h}_{1}-a{X}^{st}\right)+2{h}_{4}{X}^{st}+{h}_{5}{Z}^{st}\right\}x+\left(Wa-{h}_{4}+2c{X}^{st}+e{Z}^{st}\right){x}^{2}\\ &\quad+\left(p-b{X}^{st}-{h}_{5}{X}^{st}-d{{X}^{st}}^{2}-f{Z}^{st}\right)z-\left({h}_{5}+Wb-e{X}^{st}+f{Z}^{st}\right){x}{z}-f{X}^{st}{z}^{2},\end{aligned} $$

27 $$ \begin{aligned}\frac{dz}{dt}&=\left\{2{h}_{4}{X}^{st}+{h}_{5}{Z}^{st}-c{{X}^{st}}^{2}-e{X}^{st}{Z}^{st}\right\}x+\left({h}_{4}-2c{X}^{st}-e{Z}^{st}\right){x}^{2}+\left(-p+{h}_{5}{X}^{st}+d{{X}^{st}}^{2}+f{Z}^{st}{X}^{st}\right)z\\ &\quad+\left({h}_{5}+2d{X}^{st}-e{X}^{st}+f{Z}^{st}\right)xz+f{X}^{st}{z}^{2},\end{aligned} $$

where the coefficients *a, b, c, d, e,* and *f* (>0) represent the dependence of the diffusion process on the population density shown in *a*_*X*_ and *a*_*Z*_ in Eqs. (13) and (14), and *h*_*1*_, *h*_*4*_, and *h*_*5*_ are proportional to the diffusion coefficient, *D*_*XW*_, *D*_*XX*_, and *D*_*XZ*_.

### Numerical simulation using the data of infection numbers in Japan

**Figure 2:**
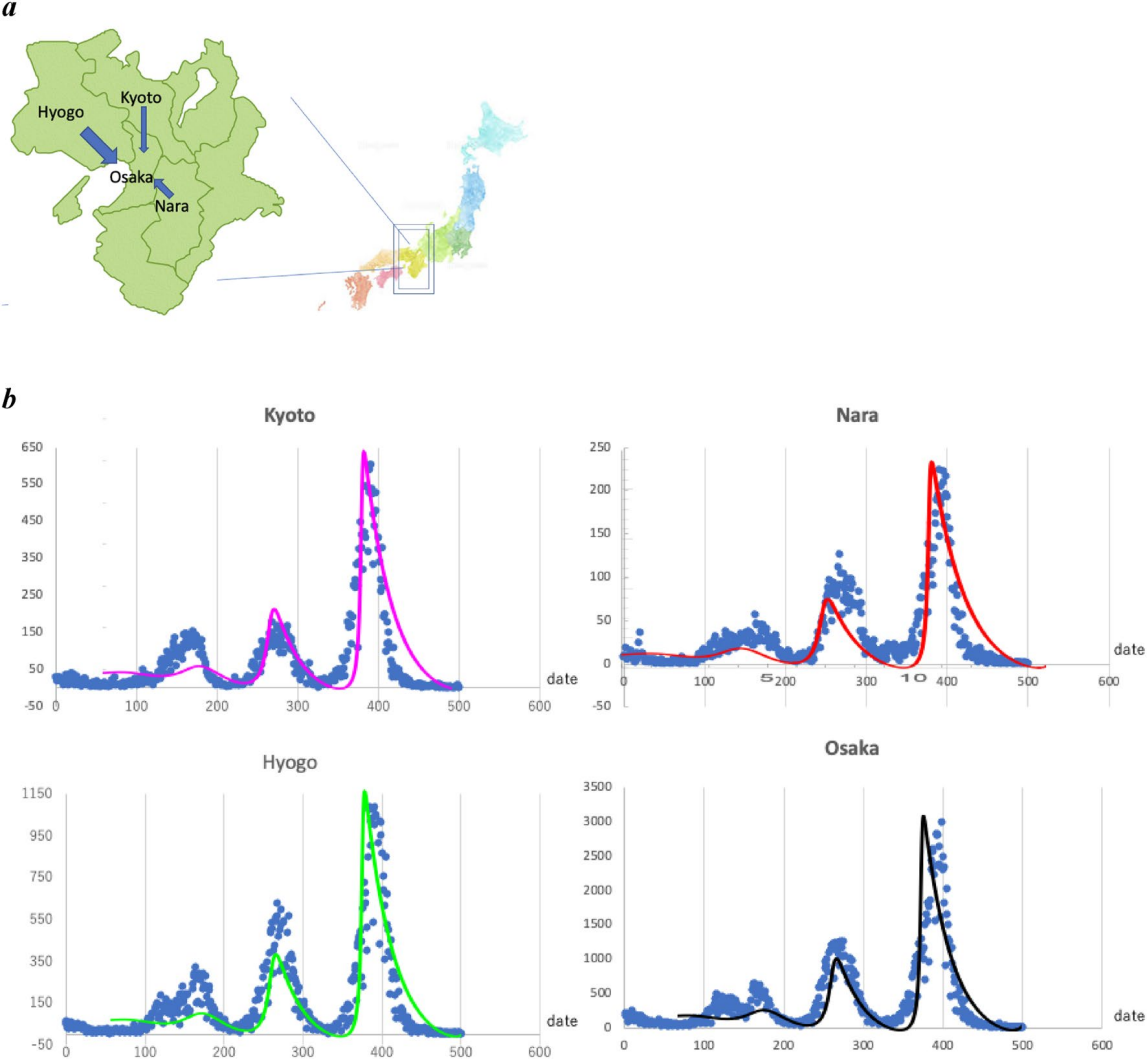

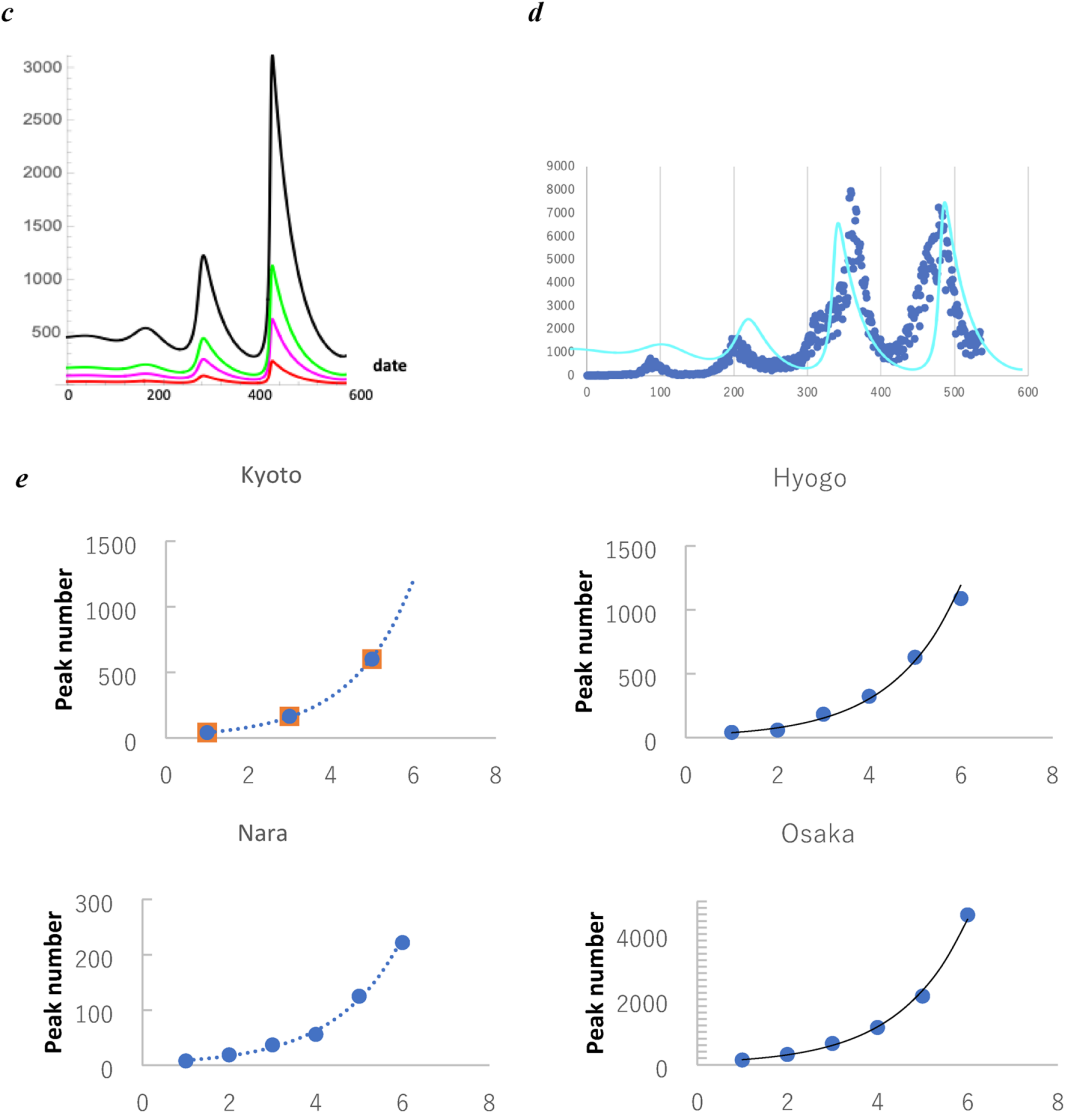
Numerical simulation of the new infection. (**a**) The maps of Japan and the Kansai region are open resources provided freely (https://frame-illust.com/?p=10227; https://imagenavi.jp/search/detail.asp?id=31596606). (**b**) Simulation of the newly infected population. The blue circle plot shows the infection number in the Kansai region in Japan (from January 2020 to July 2022). The horizontal axis represents the date from the 200th date of the infection report, and the vertical axis represents the new infection number, respectively. The theoretical plot was fit to the actual maximum and minimal infection number of the plot. *W* = 1.0 and *p* = 1.0 for Kyoto (pink), Nara (red), Hyogo(green), and Osaka(black). The simulation calculation was performed using the Mathematica cord (See method, Mathematica was ver 12), shown in the Kansai region. (**c**) The marged four theoretical simulated curves. The horizontal axis represents the date from the 200th date of the infection report. (**d**) The simulated infectious wave oscillation in all of Japan. *W* = 0.9 and *p* = 1.0 were used. The line colours are light blue. The blue circle plot shows the new infection number. (**e**) The peak of infected number in each prefecture. The vertical axis represents the new infection number, and the horizontal axis represents the number of the peak.

In Japan, the numbers of new cases of infection have been reported daily in each prefecture (Supplement Data). The daily reporting has been initiated since February 2020. Two hundred days after the onset of the infection, waves are observed, and the amplitude of the oscillations has become greater over time. Analysing this data is valuable in deciding on infection control measures. We applied a model shown in Eqs. (26) and (27) for the Kansai region based on metapopulation model^7,12,20,21^. The network of the Kansai region (population of 18 million, the second largest area in Japan) in the current simulation, consisting of nodes representing Osaka, Kyoto, Hyogo, and Nara prefectures, was assumed. Kansai region forms a metropolitan economic zone around Osaka, and the number of infected people was expected to be synchronized. There is a movement to Osaka prefectures from Hyogo, Nara, and Kyoto prefectures, while there is little movement between Hyogo, Nara, and Kyoto (Fig. 2a). Herein, infection propagation during transport was not considered. We estimated a daily net transfer term to Eqs. (26) and (27), in which *g*_*1*_* x*_*1*_*, g*_*2*_* x*_*2*_*,* and *g*_*3 *_*x*_*3*_ represent the transport number of exposed population in Kyoto, Nara, and Hyogo, respectively, where the transfer coefficients are given by *g*_*1*_ (= 5.0 × 10^–5^)*, g*_*2*_ (= 1.0 × 10^–4^)*,* and *g*_*3*_ (= 1.0 × 10^–4^), respectively. The differential equations for newly infected population were the same over the whole Kansai region. The kinetic equations of *X*_*i*_ and *Z*_*i*_ (*i* = 1, 2, 3, 4: Kyoto, Nara, Hyogo, Osaka) are written as follows:

28 $$ \begin{aligned}\frac{d{x}_{1}}{dt}&=-\left\{W\left({h}_{1}-a{X}^{st}\right)+2{h}_{4}{X}^{st}+{h}_{5}{Z}^{st}\right\}{x}_{1}+\left(Wa-{h}_{4}+2c{X}^{st}+e{Z}^{st}\right){{x}_{1}}^{2}\\ &\quad+\left(p-b{X}^{st}-{h}_{5}{X}^{st}-d{{X}^{st}}^{2}-f{Z}^{st}\right){z}_{1}-\left({h}_{5}+Wb-e{X}^{st}+f{Z}^{st}\right){x}_{1}{z}_{1}-f{X}^{st}{{z}_{1}}^{2}-{g}_{1}{x}_{1},\end{aligned} $$

29 $$ \begin{aligned}\frac{d{x}_{2}}{dt}&=-\left\{W\left({h}_{1}-a{X}^{st}\right)+2{h}_{4}{X}^{st}+{h}_{5}{Z}^{st}\right\}{x}_{2}+\left(Wa-{h}_{4}+2c{X}^{st}+e{Z}^{st}\right){{x}_{2}}^{2}\\ &\quad+\left(p-b{X}^{st}-{h}_{5}{X}^{st}-d{{X}^{st}}^{2}-f{Z}^{st}\right){z}_{2}-\left({h}_{5}+Wb-e{X}^{st}+f{Z}^{st}\right){x}_{2}{z}_{2}-f{X}^{st}{z}^{2}{-{g}_{2}x}_{2},\end{aligned} $$

30 $$\begin{aligned}\frac{d{x}_{3}}{dt}& =-\left\{W\left({h}_{1}-a{X}^{st}\right)+2{h}_{4}{X}^{st}+{h}_{5}{Z}^{st}\right\}{x}_{3}+\left(Wa-{h}_{4}+2c{X}^{st}+e{Z}^{st}\right){{x}_{3}}^{2}\\ &\quad+\left(p-b{X}^{st}-{h}_{5}{X}^{st}-d{{X}^{st}}^{2}-f{Z}^{st}\right){z}_{3}-\left({h}_{5}+Wb-e{X}^{st}+f{Z}^{st}\right){x}_{3}{z}_{3}-f{X}^{st}{{z}_{3}}^{2}{-{g}_{3}x}_{3},\end{aligned}$$

31 $$\begin{aligned}\frac{d{x}_{4}}{dt}& = -\left\{W\left({h}_{1}-a{X}^{st}\right)+2{h}_{4}{X}^{st}+{h}_{5}{Z}^{st}\right\}{x}_{4}+\left(Wa-{h}_{4}+2c{X}^{st}+e{Z}^{st}\right){{x}_{4}}^{2} \\ &\quad +\left(p-b{X}^{st}-{h}_{5}{X}^{st}-d{{X}^{st}}^{2}-f{Z}^{st}\right){z}_{4}-\left({h}_{5}+Wb-e{X}^{st}+f{Z}^{st}\right){x}_{4}{z}_{4}\\ &\quad-f{X}^{st}{{z}_{4}}^{2}{+{g}_{1}x}_{1}{+{g}_{2}x}_{2}{+{g}_{3}x}_{3},\end{aligned}$$

32 $$\begin{aligned}\frac{d{z}_{1}}{dt}& = \left\{2{h}_{4}{X}^{st}+{h}_{5}{Z}^{st}-c{{X}^{st}}^{2}-e{X}^{st}{Z}^{st}\right\}{x}_{1}+\left({h}_{4}-2c{X}^{st}-e{Z}^{st}\right){{x}_{1}}^{2}+\left(-p+{h}_{5}{X}^{st}+d{{X}^{st}}^{2}+f{X}^{st}{Z}^{st}\right){z}_{1}\\ &\quad+\left({h}_{5}+2d{X}^{st}-e{X}^{st}+f{Z}^{st}\right){x}_{1}{z}_{1}+f{X}^{st}{{z}_{1}}^{2},\end{aligned}$$

33 $$ \begin{aligned}\frac{d{z}_{2}}{dt}&=\left\{2{h}_{4}{X}^{st}+{h}_{5}{Z}^{st}-c{{X}^{st}}^{2}-e{X}^{st}{Z}^{st}\right\}{x}_{2}+\left({h}_{4}-2c{X}^{st}-e{Z}^{st}\right){{x}_{2}}^{2}\\ &\quad+\left(-p+{h}_{5}{X}^{st}+d{{X}^{st}}^{2}+f{X}^{st}{Z}^{st}\right){z}_{2}+\left({h}_{5}+2d{X}^{st}-e{X}^{st}+f{Z}^{st}\right){x}_{2}{z}_{2}+f{X}^{st}{{z}_{2}}^{2},\end{aligned} $$

34 $$ \begin{aligned}\frac{d{z}_{3}}{dt}&=\left\{2{h}_{4}{X}^{st}+{h}_{5}{Z}^{st}-c{{X}^{st}}^{2}-e{X}^{st}{Z}^{st}\right\}{x}_{3}+\left({h}_{4}-2c{X}^{st}-e{Z}^{st}\right){{x}_{3}}^{2}\\ &\quad+\left(-p+{h}_{5}{X}^{st}+d{{X}^{st}}^{2}+f{Z}^{st}{X}^{st}\right){z}_{3}+\left({h}_{5}+2d{X}^{st}-e{X}^{st}+f{Z}^{st}\right)xz+f{X}^{st}{{z}_{3}}^{2},\end{aligned} $$

35 $$ \begin{aligned}\frac{d{z}_{4}}{dt}&=\left\{2{h}_{4}{X}^{st}+{h}_{5}{Z}^{st}-c{{X}^{st}}^{2}-e{X}^{st}{Z}^{st}\right\}{x}_{4}+\left({h}_{4}-2c{X}^{st}-e{Z}^{st}\right){{x}_{4}}^{2}\\ &\quad+\left(-p+{h}_{5}{X}^{st}+d{{X}^{st}}^{2}+f{X}^{st}{Z}^{st}\right){z}_{4}+\left({h}_{5}+2d{X}^{st}-e{X}^{st}+f{Z}^{st}\right){x}_{4}{z}_{4}+f{X}^{st}{{z}_{4}}^{2},\end{aligned} $$

The least value, 5.0 × 10^–5^, was assigned to *g*_*1*_, because the transport from Kyoto to Osaka is less than that of other prefectures to Osaka (https://www2.city.kyoto.lg.jp/sogo/toukei/Population/Dotai/). The numerical calculation was performed over a sufficiently long period to evaluate the trend in system behaviour using a Mathematica cord, and the newly infectious number wavy oscillation was well simulated (Fig. 2b). The kinetic parameters are shown in Table 1.

**Table 1:** Parameters for simulation.

	Notation	Kansai except Kyoto	Kyoto	All Japan
*h*_*1*_	Proportional to diffusion coefficient of *X*	0.12	0.12	0.14
*h*_*2*_	Proportional to diffusion coefficient of *W*	0.004	0.004	0.004
*a*	Proportional to ∂*h*_*1*_ /∂*x*	1000	1000	1000
*b*	Proportional to ∂*h*_*1*_ /∂z	1000	1000	1000
*c*	Proportional to ∂*h*_*4*_ /∂x	105	105	105
*d*	Proportional to ∂*h*_*4*_ /∂z	105	105	105
*e*	Proportional to ∂*h*_*5*_ /∂x	10	10	10
*f*	Proportional to ∂*h*_*5*_ /∂z	10	10	10
*D*_*XX*_	Interaction kinetic coefficient of *X* and *X*	155	155	155
*D*_*XZ*_	Interaction kinetic coefficient of *X* and *Z*	155	155	155
*W*	Cluster size	1.0	1.0	0.9
*p*	Recovery rate	1.0	1.0	1.0
*g*_*1*_	Movement rate from Kyoto to Osaka	0.00005		
*g*_*2*_	Movement rate from Nara to Osaka	0.0001		
*g*_*3*_	Movement rate from Hyogo to Osaka	0.0001		

According to the new infection report, the date showing the peak of the newly infected numbers in Osaka tended to lag behind the dates showing the peaks in other prefectures. The model predicted the lag in the infection numbers (Fig. 2c). Additionally, although the total infection number in Japan was well simulated by altering *W* from 1.0 to 0.9, the Eqs. (26) and (27) simulated the wavey oscillation in the same manner (Fig. 2d). Besides, the peak of the infection number in each wavy oscillation is approximated by an exponential function of which the exponent of the Napier number is nearly 0.67; then, each function is proportional to 1.95^t^, showing an increase of approximately 2.0 (~ e^0.67^) times at each infectious wave (Fig. 2e).

### Recovery rate factor as a critical order parameter

**Figure 3:**
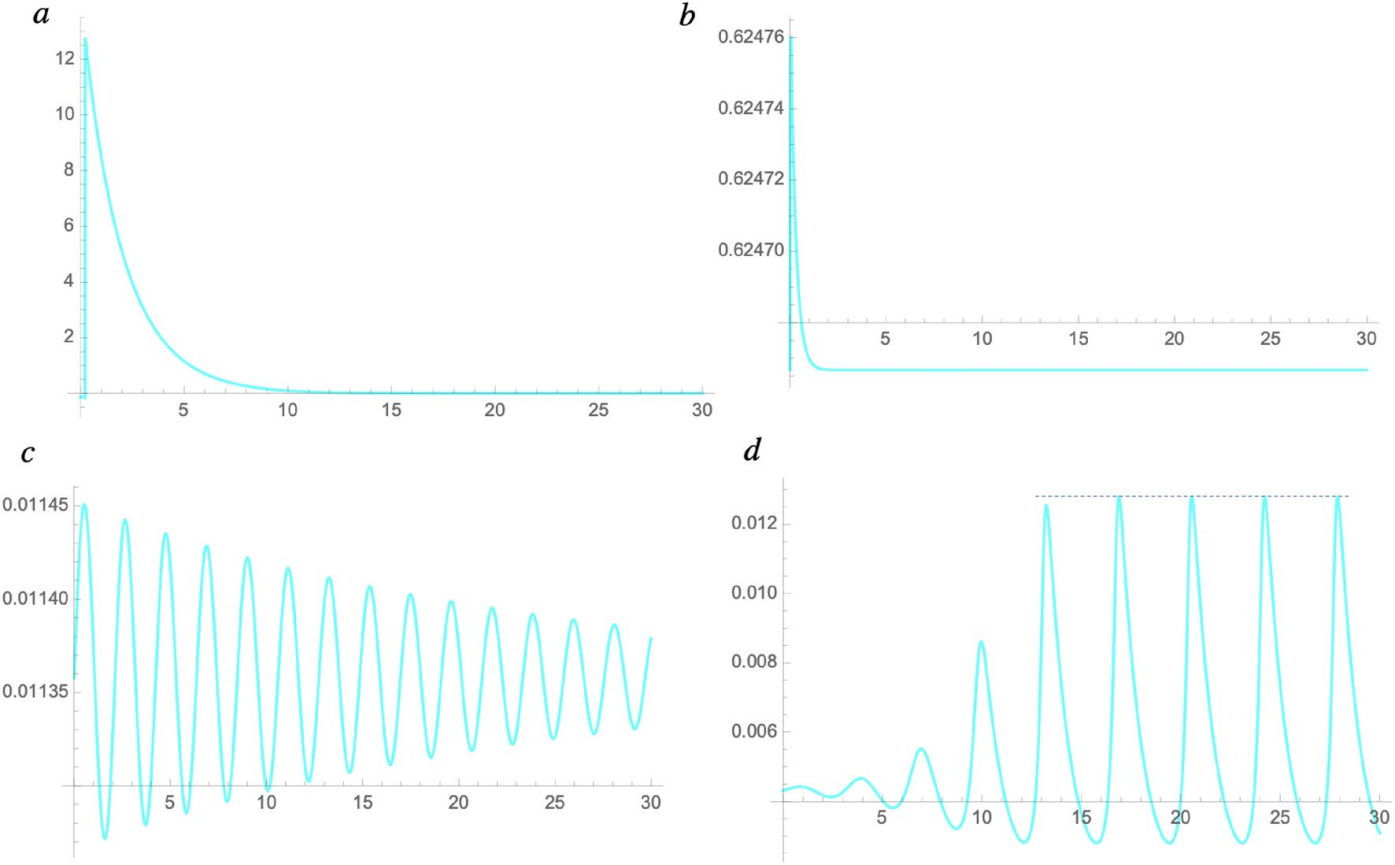
Recovery rate factor and infectious wave. The recovery rate coefficient *p* is the first order term of *z*_*1*_ in the right-hand side in Eq. (32). The simulation plots show the relative value corresponding to the new infection number in Kyoto when (**a**) *p* = 0.5, (**b**) *p*_*c*_ = 0.52, (**c**) 0.7, and (**d**) 1.0. (**a**) shows the infection explosion. (**c**) and (**d**) show the simulated infection wave. (**d**) shows that the amplitude approaches a plateau, as indicated by the dotted line. Scales at the horizontal axes represent the relative value, and one scale interval represents 30 days. Scales at the vertical axes represent the relative value corresponding to the infection number when the initial value was set to 10^–4^ for the simulation (see method, “Numerical simulation”).

**Figure 4:**
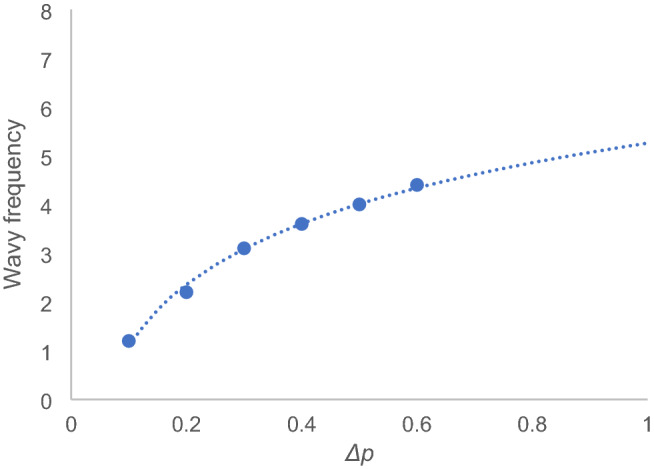
Plot of the mean frequency of the simulated infectious wave for Δ*p* . The fitting line is the result of regression analysis on the logarithmic function. The correlation coefficient was > 0.95. The vertical y-axis represents the wavy frequency given by the relative value, and the horizontal axis represents the recovery rate difference *Δp* = *p* − *p*_*c*_. The vertical axis represents the frequency per the time unit (30 days) in the horizontal axis in Fig. 3.

Subsequently, we varied the recovery rate factor *p* and simulated the infection wavy oscillation (Fig. 3). For simplification, we simulated using the kinetic Eqs. (26) and (27) without the transport term *g*_*i*_* xi*. We set *h*_*1*_ = 0.12 (Table 1). The vertical axes represent relative value corresponding to the new infection number in Kyoto prefecture. Particularly, at the threshold *p*_*c*_ = 0.52, the recovery rate coefficient changes from positive to negative. Correspondingly, at *p* < 0.52, a significant increase in the infection number occurs; as the plot in Fig. 3a shows, the relative values peaked rapidly; however, when the threshold of the recovery factor *p*_*c*_ is exceeded, the infection explosion is suppressed, and the infection number shows a wavy oscillation as shown in Fig. 3d. This  simulation result indicates that an explosion of infections can quickly occur when a sufficient recovery rate cannot be ensured due to the collapse of the medical system. Subsequently, the oscillation frequency was calculated using the wave number per 30 days. Table 1 shows the coefficients used in the simulation. In this plot in Fig. 3d, the wavy oscillation approaches a plateau as shown by a dotted line, indicating that infection is controlled. Subsequently, the relation between *Δp* = *p* − *p*_*c*_ and the wavy oscillation period was analysed. When *p* exceeded the critical value (*p*_*c*_ = 0.52), a wavy oscillation was simulated, and the period increased. The reciprocal of the wave number per 30 days in the obtained plot was taken as the frequency. The plot data indicates a relationship between the mean wavy frequency of the simulated wavy oscillation of the infection number and *Δp*. As a result, the frequency was observed as a logarithmic function of *Δp* (Fig. 4):

36 $$f=1.8\, \mathrm{log}\Delta{p} +5.3.$$

We found that when the recovery rate increases in this way, the period of the infection number wavy oscillation becomes longer. Thus, it is necessary to enhance the medical care system for the recovery of the infected people.

### Diffusion coefficient as a critical order parameter

**Figure 5:**
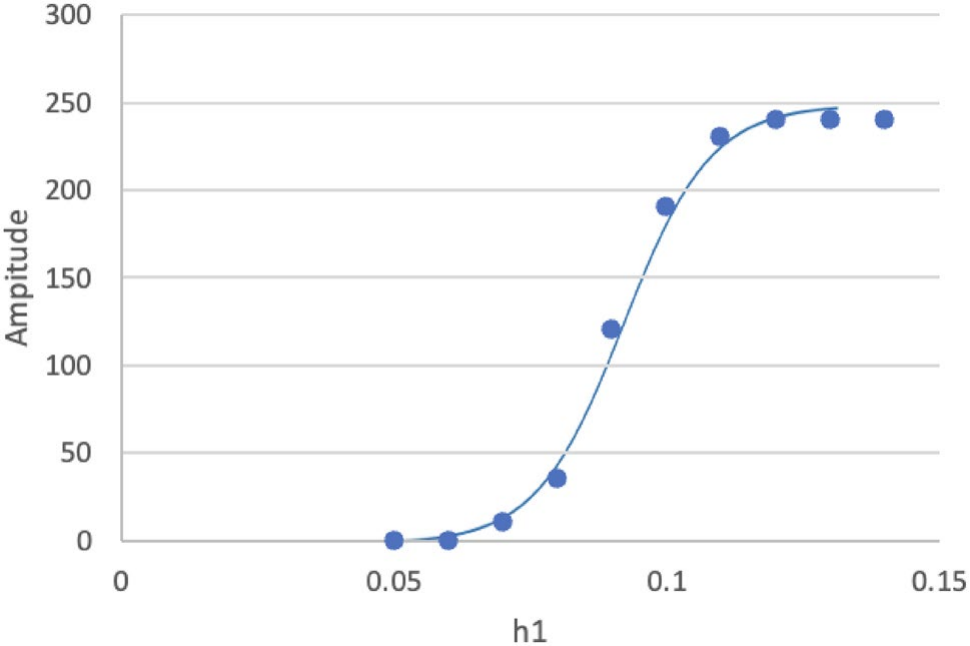
Plot of the amplitude of the infection wavy oscillation. The wavy amplitude of the infection number is plotted in reference to the diffusion coefficient *h*_*1*_. The vertical scale represents the date from the first day of the infection report. The line is fitted to a sigmoid function to the plot using the fitting algorithm of Mathematica.

The amplitude of the infection wavy curve reached a plateau nearly (*t* > 300th date in Fig. 3d). The amplitude at the plateau regarding diffusion coefficient *h*_*1*_, was plotted as a sigmoid-like curve (Fig. 5). At *h*_*1*_ < *h*_*1c*_ = 0.078, the new infectious wave was not observed. At *h*_*1*_ > *h*_*1c*_, the wavy oscillation was observed, and the amplitude value approaches the plateau, when *h*_*1*_ was near 0.12 (Table 1). Thus, individual diffusion suppression of the infected individual is critical for the infection control.

## Discussion

The infectious wavy oscillation is a unique phenomenon in Japan that has been observed for 2 to 3 years. To explain this phenomenon, we assumed that the diffusion of individuals, exposed and infectious, is proportional to their density gradient and that their diffusion coefficient is density-dependent. Our results are summarised as follows: (i) The nonlinear kinetic equations simulated the infection wavy oscillation; (ii) The recovery rates are critical for predicting whether the infection wave can be observed and whether the infection may explode; (iii) the diffusion suppression is critical for infection control. In particular, the simulation in Fig. 3 demonstrated that securing and supporting medical resources for the infectious individual's recovery is critical^22^, because a minimal decrease in the resources triggers a rapid increase in the infection number.

Infection numbers in each prefecture within the Kansai metropolitan region and all of Japan were well simulated by changing only the cluster number and diffusion coefficient. In this way, the necessary change in the simulation was minimal, suggesting that the current model is available for other area in Japan. Because daily transportation is possible between the prefectures in the Kansai region, there was coordination among public policies, such as restraining the flow of individuals through communication between governors in local governments in an integrated manner within this area^7,12,22^.

In the current study, we considered new infectious number fluctuations over longer time scales where transient movement can be neglected. The change in the number of people by the transfer can be represented by the difference between those moving to and from the node (prefecture), mainly moving to and from Osaka (the central node of the Kansai network). Because migration is limited to daily travelling for business, the difference can be nearly equal to zero and the transfer coefficient *g*_*i*_ is sufficiently small. For these reasons, we omitted the new infection during the transmission during daily transportation.

Moreover, the infected number in all of Japan was simulated using a cluster value of *W* = 0.9. Therefore, the cooperation of the four prefectures in the Kasai region is unique (*W* = 1.0) and relatively independent of the kinetics in all of Japan. Because Japan is an island country, there are restrictions on the flow of people. We may see independent kinetics like the Kansai region unfold in each region. Hence, it may be challenging to construct a simulation model common to all of Japan. However, the oscillatory phenomena reported here have been observed nationwide and similar mdelling will be possible for the infection number simulation in each region.

In conclusion, the new infection number model based on nonlinear diffusion kinetics can well predict the wavy oscillation of the number of infected people. This modelling may provide essential insights into similar transmissions in the future.

## Methods

### New infection number database

The number of new infections in Japan is according to a database published by https://www.mhlw.go.jp/stf/seisakunitsuite/bunya/0000121431_00086.html.

### Numerical simulation

A simulation was performed using Mathematica® version 12 (Wolfram Research, Champaign, IL, USA). In the case that h1 = 0.12, h2 = 0.0004, a = 1000, b = 1000, c = 105, d = 105, e = 10, f = 10, p = 1.0, Dxx = 155, Dxz = 155, W = 0.9, X = 2/h1, Z = (2 (h1^2 W + Dxx 2))/(h1 (h1 p − Dxz 2)), g1 = 0.0005, Mathematica cord for plotting of infected persons in Kyoto is as follows:

h1 = 0.12, h2 = 0.0004, a = 1000, b = 1000, c = 105, d = 105, e = 10, f = 10, p = 1.0, Dxx = 155,

Dxz = 155, W = 1, X = 2/h1, Z = (2 (h1^2 W + Dxx 2))/(h1 (h1 p − Dxz 2)), g1 = 0.00005.

NDSolve[{x'[t] == − (W (h1 − a X) + 2 X Dxx + Dxz Z) x[t] + (W a − Dxx + 2 c X + e Z) x[t]^2 + (p − Dxz X − b X − d X^2 − f X Z) z[t] −  (Dxz + W b − e X + f Z) x[t] z[t] − (f X) z[t]^2- g1 × 1[t]

z'[t] == (2 X Dxx + Dxz Z − c X^2 − e X Z) x[t] + (Dxx − 2 c X − e Z) x[t]^2 + (Dxz + 2 X d − e X + f Z) x[t] z[t] + (Dxz X − p + d X^2 + f X Z) z[t], x[0] == 0.0001, z[0] == 0.0000}, {x, z}, {t, 0, 10,000}, MaxSteps→50000], g = Plot[{Z + z[t]}/. %%, {t, 0, 20}, PlotRange→All, PlotStyle→{RGBColor[0, 1, 1]}, PlotRange→All].

In the above, *t* = 10 corresponds to 400 days. For Nara, Hyogo, and Osaka. The extremum of the theoretical plot was fitted to match the extremum of the number of infectious individuals by producing 601 × 70, 220 × 46, 1088 × 46, 3004 × 46 with *z*_1_, *z*_2_, *z*_3_, and *z*_4_ in (Fig. 2b). These 601, 220, 1088, and 3004 were the maximum number of the new infected. In addition, the dates of the maxima and minima values of the theoretical plot were adapted so that the infection number coincided with the dates on which the infection number took the local maxima and local minima.

The actual new infection numbers in Kyoto, Nara, Hyogo, and Osaka were calculated by producing 601 × 70, 220 × 46, 1088 × 46, 3004 × 46 with the value of *Z*_*1*_*, Z*_*2*_*, Z*_*3*_, and *Z*_*4*_.

## Supplementary Information

Supplementary Information.

## Data Availability

The datasets generated and/or analysed during the current study (See Supplement data) are available as supplemental data sets. In particular, the infection number is updated daily in the shown (https://www.mhlw.go.jp/stf/seisakunitsuite/bunya/0000121431_00086.html.)
